# Microarray Analysis of lncRNA and mRNA Reveals Enhanced Lipolysis Along With Metabolic Remodeling in Mice Infected With Larval *Echinococcus granulosus*

**DOI:** 10.3389/fphys.2020.01078

**Published:** 2020-08-21

**Authors:** Yang Lu, Hua Liu, Xiao-ying Yang, Jia-xue Liu, Meng-yu Dai, Jia-cheng Wu, Yu-xin Guo, Tian-cheng Luo, Fen-fen Sun, Wei Pan

**Affiliations:** ^1^Jiangsu Key Laboratory of Immunity and Metabolism, Department of Pathogenic Biology and Immunology, Xuzhou Medical University, Xuzhou, China; ^2^Department of Clinical Medicine, Xuzhou Medical University, Xuzhou, China; ^3^Key Laboratory of Parasite and Vector Biology, Ministry of Health, National Institute of Parasitic Diseases, Chinese Center for Disease Control and Prevention, Shanghai, China

**Keywords:** *Echinococcus granulosus*, protoscoleces, adipocyte, adipogenesis, long non-coding RNA, microarray analysis

## Abstract

Parasitic infection improves metabolic homeostasis in “western diet”-induced obesity through the regulation of adipogenesis. However, the underlying mechanism is not yet fully understood. Using microarray analysis, this study investigated the long non-coding RNA (lncRNA) and messenger RNA (mRNA) profiles of subcutaneous adipose tissues from mice infected with *Echinococcus granulosus* protoscoleces. A total of 1052 mRNA (541 upregulated, 511 downregulated) and 220 lncRNA (126 upregulated, 94 downregulated) transcripts were differentially expressed (fold change ≥2, *P* < 0.05) in the infected subcutaneous adipose tissues. When compared with the control group, the infected mice showed a decrease in adipose tissue mass and a reduction in adipocyte size. Indirect calorimetry revealed the change in the energy metabolism after infection, characterized by a lower CO_2_ production and O_2_ consumption, a sharp decline in carbohydrate oxidation, and a slight increase in fat oxidation. Gene Ontology and Kyoto Encyclopedia of Genes and Genomes pathway analyses showed that the parasitic infection reprogrammed a complex metabolic network. Notably, “lipoxygenase” and “arginine and proline metabolism” pathways were significantly upregulated while “glycolysis,” “tricarboxylic acid cycle,” “*de novo* lipogenesis,” and “lipid droplet” pathways were dramatically downregulated. In addition, several key lncRNAs were associated with insulin resistance and adipocyte differentiation. Overall, the present study suggested that *E. granulosus* infection could enhance lipolysis. Thus, our findings provide novel insights into parasite-mediated metabolic homeostasis, and into the mechanism of hypertrophic adipocytes in obesity.

## Introduction

Parasites pose a threat to human health since they are widely considered as agents of disease and death (Hellard et al., 2015). Following infection, these organisms can compete with the host for nutrients, while also regulating its metabolism and immune system. Parasites have developed refined machinery to evade the host’s immune system by manipulating its response (Gomes et al., 2016). It is well known that T-helper type 2 (Th2) responses are central to parasitic infections, and can participate in regulation of host’s immune system (Affinass et al., 2018). Th2 responses can create an anti-inflammatory immune microenvironment and improve host metabolic disorders (van der Zande et al., 2019). Such immunological characteristics make parasites and their derived molecules attractive models for the treatment of inflammatory and metabolic disorders. For example, *Trichuris muris* infection was shown to protect mice deficient in *NOD2* (susceptibility gene for Crohn’s disease) from Th2 response-dependent intestinal abnormalities (Ramanan et al., 2016). Additionally, the excreted or secreted molecules-62 (ES-62) derived from *Acanthocheilonema viteae* (a filarial nematode parasite of gerbils) are anti-inflammatory by virtue of covalently attached phosphorylcholine moieties (Doonan et al., 2019). The subcutaneous administration of this immunomodulator was recently reported to protect against collagen-induced arthritis (Doonan et al., 2019).

Currently, obesity is a main public health concern since obese individuals are at a greater risk of developing chronic disease and often present clinical parameters of metabolic syndrome (MetS), insulin resistance, and systemic markers of chronic low-grade inflammation (Alberti et al., 2005; Andersen et al., 2016; Affinass et al., 2018). Adipose tissue accumulation is a common clinical phenomenon in obese individuals (Gijs, 2017). Adipose tissue is the main lipid storage depot in the human body and, as such, plays a crucial role in the regulation of dietary fat daily influx into circulation (Andersen et al., 2016; Jais and Brüning, 2017). However, in obesity, the adipose tissue may not expand properly when there is a need to store the energy surplus. In turn, this may lead to ectopic fat deposition in tissues involved in metabolic homeostasis and, consequently, insulin resistance and MetS (Feakins, 2016). Thus, adipogenesis is an essential event in obesity and MetS development.

An increasing number of studies have shown that parasitic infection can regulate adipogenesis. The intestinal nematode parasite *Heligmosomoides polygyrus* was reported to attenuate obesity in high-fat diet-induced obese mice (Affinass et al., 2018). The infected individuals exhibited a markedly upregulated expression of uncoupling protein 1 (a key protein involved in energy expenditure) in the adipose tissue, suppression of glucose and triglyceride levels, and alteration in the expression of key genes involved in lipid metabolism (Su et al., 2018). *Trypanosome brucei* is a parasite that uses the adipose tissue as a niche, enhancing fatty acid β-oxidation and causing weight loss in model mice (Trindade et al., 2016). These studies suggested that helminths may play a protective role against MetS (Crowe et al., 2017) and that exploring the underlying mechanism of adipogenesis in a parasitic infection context may provide a novel strategy for the treatment of obesity and MetS.

*Echinococcus granulosus* (*E. granulosus*) is a known cestode with high host adaptability and worldwide distribution. After oral uptake of the eggs by intermediate hosts, the activated oncospheres hatch in the gastrointestinal tract, where they penetrate the intestinal wall and enter the bloodstream. Eventually, the parasites reach the visceral organs (such as the liver, lung, heart, and kidney), where they develop into hydatid cysts. *E. granulosus* can infect many intermediate hosts and go unnoticed for several decades, since it has evolved strategies to subvert the immune responses (Peón et al., 2016). Previous studies have focused on the immunoregulatory role of *E. granulosus* and its derived molecules (Pan et al., 2013, 2018; Díaz, 2017). However, their metabolic regulatory activity of *E. granulosus* and its derived molecules has been largely neglected.

Long non-coding RNAs (lncRNAs) are emerging as important regulators of cellular signaling and gene expression in numerous cell types. These molecules are long RNA transcripts (>200 bp) that lack protein-coding ability (Ferrè et al., 2016). lncRNAs can regulate cell function through a variety of mechanisms (Sun and Lin, 2019). For instance, they can function as scaffolds to bring two or more proteins into a functional ribonucleoprotein complex. lncRNAs can also act as decoys to titrate a protein away from its original target, as guides to recruit chromatin modification enzymes to specific loci on chromosomes, and as microRNA sponges to buffer the inhibitory functions of microRNAs on gene expression. Recent studies have revealed an extensive list of regulators that govern adipocyte differentiation, and the scope of these regulators has been greatly expanded to lncRNAs. For example, the very recently identified adipogenic lncRNA Plnc1 was found to promote differentiation into mature adipocytes (Sun et al., 2019). However, the understanding of the underlying mechanisms of lncRNA in adipocyte regulation is still in its infancy.

The present study investigated whether the larval *E. granulosus* infection in mice could regulate adipogenesis and determined the differential expression profiles of both mRNA and lncRNA. The results showed that the fat mass and adipocytes’ size were significantly decreased, following infection. Moreover, several differentially expressed lncRNAs and mRNAs were found to regulate the metabolic reprogramming and differentiation of adipocytes. These results provided a novel insight into host-parasite interplay and several clues for the treatment of obesity and its associated Mets.

## Materials and Methods

### Ethics Approval

This study was carried out in strict accordance with the recommendations of the Guide for the Care and Use of Laboratory Animals of the Ministry of Health, China. The protocol was approved by the Laboratory Animal Welfare and Ethics Committee (LAWEC) of Xuzhou Medical University (Xuzhou, China, SCXK (Su) 2015-0009). All surgery was performed under sodium pentobarbital (100 mg/kg) anesthesia and all efforts were made to minimize suffering.

### Mice and Parasites

Female C57BL/6J mice (6–8 weeks old) were obtained from Shanghai Laboratory Animal Center (SLAC, Shanghai, China) and were bred in the university facilities. All mice were fed by the experimental animal growth and reproduction diet (Amufi company, Suzhou, China) containing 20% protein, 4% lipid and 5% carbohydrate, and were housed in an air-conditioned room at 24°C with a 12 h dark/light cycle and permitted free access to food and water.

The protoscoleces of *E. granulosus* (EgPSC) were obtained from the hydatid cysts of naturally infected sheep livers under aseptic conditions, and washed three times using 0.9% NaCl containing 100 mg/ml penicillin and 100 U/ml streptomycin. Female C57BL/6J mice were intraperitoneally injected with a 200 μl suspension containing 2000 live EgPSC in 0.9% NaCl to establish the Eg model, and the control mice were received 200 μl 0.9% NaCl (Pan et al., 2013, 2018; Yu et al., 2018). The infected and control mice were sacrificed for experiment after 7 months of infection.

### Indirect Calorimetry

The profile of systemic energy metabolism was determined by indirect calorimetry using the Oxymax CLAMS system (Columbus Instruments) at 7^th^ month post infection. Mice were housed individually in CLAMS cages and allowed to acclimate for 24 h (12 h day and 12 h night) in 25°C with unrestricted excess to food and water. O_2_ consumption (VO_2_), CO_2_ release (VCO_2_), Respiratory Exchange Ratio (RER) and food intake were recorded for 24 h spanning a single light-dark cycle. Carbohydrate oxidation was calculated using the formula [(4.585^∗^VCO_2_)–(3.226^∗^VO_2_)]^∗^4, in which the 4 represents the conversion from mass per time unit to kcal per time unit (Péronnet and Massicotte, 1991). Similarly, fat oxidation was calculated using the formula [(1.695^∗^VO_2_)-(1.701^∗^VCO_2_)]^∗^9 (Péronnet and Massicotte, 1991).

### Glucose Tolerance Test (GTT)

Glucose tolerance test was performed at 7^th^ month post-infection. After fasting for 16 h, mice were given a glucose solution in the abdominal cavity at a dose of 1 g/kg. Blood samples were collected at 0, 30, 60, 90, and 120 min after injection, and blood glucose test paper was used to detect blood glucose. The curve of blood glucose over time was drawn by Graphpad 6 software and the total area under the curve (AUC) was calculated.

### Serum Biochemical Indices

After sacrifice, the blood samples from mice eyeballs were centrifuged in 12,000 rpm, 4°C for 20 min. The serum concentration of aspartate aminotransferase (AST), alanine aminotransferase (ALT), triglyceride, cholesterol, high density lipoprotein (HDL) and low density lipoprotein (LDL) was measured by automatic biochemical analyzer (Roche Cobas 701, Roche Diagnostics Ltd.).

### Histopathological Examination

Adipose tissue was immersed in 4% paraformaldehyde for 4 h, and transferred to 70% ethanol. Individual lobes of adipose tissue were placed in processing cassettes, dehydrated through a serial alcohol gradient, and embedded in paraffin wax blocks. Before immunostaining, 5 μm-thick adipose tissue sections were dewaxed in xylene, rehydrated through decreasing concentrations of ethanol, and washed in PBS. Stain the slide with 1% eosin Y solution for 10–30 s with agitation. Dehydrate the sections with two changes of 95% alcohol and two changes of 100% alcohol for 30 s each. At last, extract the alcohol with two changes of xylene (Fischer et al., 2008). After staining, sections were dehydrated through increasing concentrations of ethanol and xylene and cover with a coverslip.

### Microarray Profiling

The lncRNA and mRNA expression patterns of three EgPSC infected mice and three control mice were detected. The Agilent-085631 was used in this experiment and data analysis of the 6 samples were conducted by OE Biotechnology Co., Ltd. (Shanghai, China). Total RNA was quantified by the NanoDrop ND-2000 (Thermo Fisher Scientific, Waltham, MA, United States) and the RNA integrity was assessed using Agilent Bioanalyzer 2100 (Agilent Technologies). The sample labeling, microarray hybridization and washing were performed based on the manufacturer’s standard protocols. Briefly, total RNAs were transcribed to double strand cDNA, then synthesized into cRNA and labeled with Cyanine-3-CTP. The labeled cRNAs were hybridized onto the microarray. After washing, the arrays were scanned by the Agilent Scanner G2505C (Agilent Technologies).

### Differential Expression Analysis

The raw data were analyzed using Feature Extraction software (version 10.7.1.1; Agilent Technologies) and then normalized using percentile normalization. Probes with least one of two conditions flagged in “*P*” were chosen for further data analysis. Calculated with the *t*-test, differentially expressed lncRNAs were identified through fold change and the *P*-value. Aberrantly expressed lncRNAs and mRNAs were defined as fold change ≥2.0 and *P* < 0.05. Hierarchical clustering was performed on six mouse adipose tissue samples using Cluster 3.0 (Stanford University School of Medicine, California, United States) and TreeView 2.0 (Baryshnikova Lab, Princeton University, New Jersey, United States) to distinguish the distinguishable gene expression pattern among the samples.

### GO and KEGG Enrichment Analysis

The Gene Ontology (GO) analysis was executed to investigate the biological functions of mRNAs and lncRNAs. Biological process (BP), cellular component (CC), and molecular function (MF) were involved in the GO terms. The enrichment analysis of GO terms with *P* < 0.05 was considered significantly. Moreover, to predict the possible pathways, the Kyoto Encyclopedia of Genes and Genomes (KEGG) pathway analysis was adopted to map the possible pathways of these differentially expressed genes.

### Co-expression Network Analysis

Cytoscape version 3.5.1 (US National Institute of General Medical Sciences) was used to describe the co-expression of lncRNAs and the protein-coding genes. Correlations with *P* < 0.05 were considered statistically significant.

### Quantitative Reverse Transcription PCR

Quantitative reverse transcription-PCR (qRT-PCR) was performed to validate the microarray results. Quantification was performed with a two-step reaction process: reverse transcription (RT) and PCR. Each RT reaction consisted of 0.5 μg RNA, 2 μl of 5 × *TransScript* All-in-one SuperMix for qPCR and 0.5 μl of gDNA Remover, in a total volume of 10μl. Reactions were performed in a GeneAmp^®^ PCR System 9700 (Applied Biosystems, United States) for 15 min at 42°C, 5 s at 85°C. The 10 μl RT reaction mix was then diluted × 10 in nuclease-free water and held at −20°C. Real-time PCR was performed using LightCycler^®^ 480 II Real-time PCR Instrument (Roche, Swiss) with 10 μl PCR reaction mixture that included 1 μl of cDNA, 5 μl of 2 × *PerfectStart*^TM^ Green qPCR SuperMix, 0.2 μl of forward primer, 0.2 μl of reverse primer and 3.6 μl of nuclease-free water. Reactions were incubated in a 384-well optical plate (Roche, Swiss). The exact thermal cycler conditions were described in a previous study (Li et al., 2016). Each sample was run in triplicate for analysis. At the end of the PCR cycles, melting curve analysis was performed to validate the specific generation of the expected PCR product. The primer sequences are shown in Supplementary Table S1, which were designed in the laboratory and synthesized by TsingKe Biotech based on the mRNA sequences obtained from the NCBI database. The expression levels were calculated using the 2^–Δ^ ^Δ^ ^*Ct*^ method (Livak and Schmittgen, 2001).

### Statistical Analysis

All statistical analysis in this study was performed using the software Graphpad Prism 6.0. All measurement data were presented as the mean with standard error of the mean (SEM). Differentially expressed mRNA and lncRNAs were identified using *t*-tests. *P* < 0.05 considered as statistically significant.

## Results

### Enhanced Lipolysis After EgPSC Infection

**FIGURE 1:**
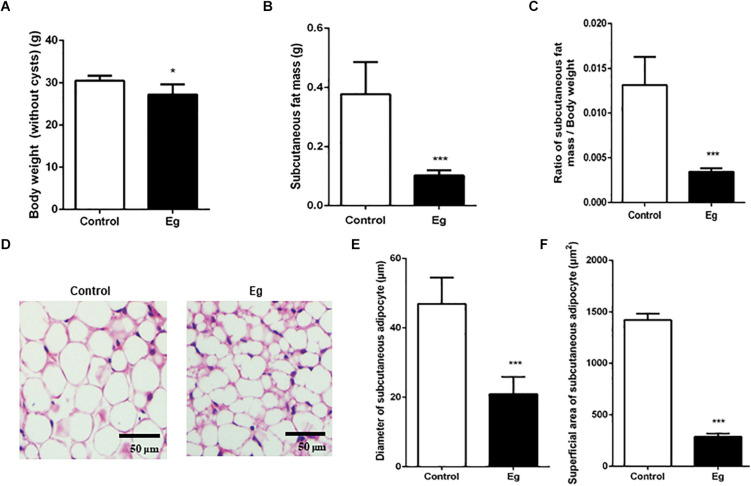
The pathological evaluation of subcutaneous fat in EgPSC infected mice. The C57BL/6J mice were sacrificed at the 7^th^ month after infection. **(A)** The body weight (without cysts). **(B)** The mass of subcutaneous fat. **(C)** The ratio of subcutaneous fat mass/body weight. **(D)** Representative adipose tissue images of hematoxylin-eosin (H&E) staining. **(E)** The statistical results of subcutaneous adipocyte diameter. **(F)** The statistical results of subcutaneous adipocyte superficial area. *n* = 8 mice for each group. The differences were analyzed using two tailed Student’s *t*-test. Data represent means with SEM. Asterisks indicate statistically significant differences compared to the control group. *P < 0.05, ***P < 0.001. ns, no significance.

There is accumulating evidence showing that parasitic infection causes pathophysiological changes in host adipose tissues (De Niz et al., 2019). However, whether this occurs following EgPSC infection remains unknown. EgPSC-infected mice gained body weight due to the cysts in their inner organs, which demonstrated the successful establishment of the infection model. After removing the cysts, the EgPSC-infected mice showed a decrease in body weight (*P* = 0.041, Figure 1A). Moreover, the mass of subcutaneous fat were significantly lower in the infected mice than in the control group (*P* < 0.001, Figure 1B). Consistent with this observation, the percentages of subcutaneous fat mass/body weight (without cysts) decreased remarkably (*P* < 0.001, Figure 1C). Hematoxylin-eosin (H&E) staining revealed shrunken adipocytes in post-infection subcutaneous fat tissue (Figure 1D). The diameter and superficial area of subcutaneous adipocytes in infected mice also decreased (*P* < 0.001, Figures 1E,F). These results suggested that EgPSC could promote lipolysis in the host. In addition, the relative expression of M1 type macrophage markers (tumor necrosis factor alpha, interleukin-6 and inducible nitric oxide synthase) was significantly downregulated (*P* < 0.05, Supplementary Figure S1A), while the relative expression of M2 macrophage markers (arginase-1 and interleukin-10) was remarkably upregulated (*P* < 0.01, Supplementary Figure S1B), which was consistent with the fact that M2 macrophages promote lipolysis (Nguyen et al., 2011).

In contrast, there was no obvious change in liver mass, triglyceride, cholesterol, low- and high-density lipoproteins after infection (*P* > 0.05, Supplementary Figures S2A–E), while serum levels of aspartate aminotransferase (AST) and alanine aminotransferase (ALT) were decreased (Supplementary Figures S2F–H). However, the parasitic infection significantly improved the mice glucose tolerance (*P* < 0.001, Supplementary Figure S3), indicating an alternative host metabolism.

### Characterization of Systemic Energy Metabolism After EgPSC Infection

The adipose tissue is the main lipid storage depot in the body and has a crucial role in burning lipids for thermogenesis (Cinti, 2018). As a result, this tissue contributes to the total energy expenditure and can modify the energy metabolism of the whole body. We hypothesized that EgPSC infection could alter the systemic energy metabolism in mice because of the evident decrease in adipose tissue mass, following infection. To corroborate this, an indirect calorimetry assay was conducted with mice housed in metabolic cages for 24 h (1 day and 1 night).

**FIGURE 2:**
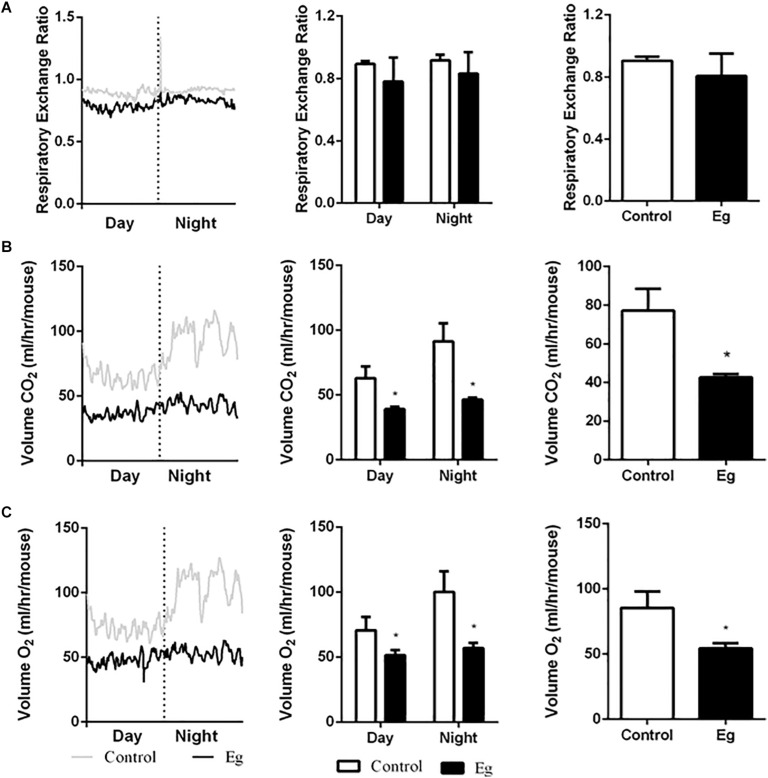
The profile of systemic energy metabolism in EgPSC infected mice. The systemic energy metabolism was determined by the indirect calorimetry. **(A)** Respiratory exchange ratio (RER). **(B)** CO_2_ release (VCO_2_). **(C)** O_2_ consumption (VO_2_). *n* = 8 mice for each group. The differences were analyzed using two tailed Student’s *t*-test. Data represent means with SEM. Asterisks indicate statistically significant differences compared to the control group. *P < 0.05.

**FIGURE 3:**
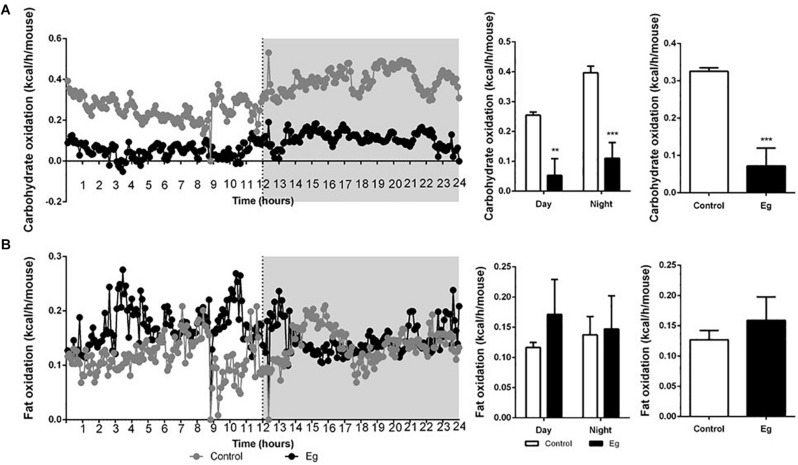
The profile of carbohydrate oxidation and fat oxidation in EgPSC infected mice. **(A)** Carbohydrate oxidation. **(B)** Fat oxidation. *n* = 8 mice for each group. Light and gray areas represent the light and dark phases, respectively. Bar graph analysis means light and dark phase results and all-time results. The differences were analyzed using two tailed Student’s *t*-test. Data represent means with SEM. Asterisks indicate statistically significant differences compared to the control group. **P < 0.01, ***P < 0.001.

Compared with the control group, the infected mice showed a comparable level of Respiratory Exchange Ratio (RER) throughout the experimental period (*P* > 0.05, Figure 2A). However, each EgPSC-infected mouse released less CO_2_ (*P* < 0.05, Figure 2B) and consumed less O_2_ (*P* < 0.05, Figure 2C). The results indicated a lower energy metabolism following infection. Moreover, a sharp decline in carbohydrate oxidation (*P* < 0.01, Figure 3A) and increased fat oxidation per mouse (*P* > 0.05, Figure 3B) were observed, suggesting that EgPSC infection could force the mice to use fatty acids as a carbon source and enhance lipid combustion (Schilperoort et al., 2018), which is consistent with fat loss following infection. However, there was no difference in food intake between the EgPSC-infected and control mice (Supplementary Figure S4), indicating that low appetite was not the cause of the observed metabolic changes.

### Differentially Expressed mRNAs and lncRNAs in Adipose Tissues After EgPSC Infection

Microarray analysis was performed to identify the pathophysiological events of EgPSC infection and the mechanism of adipogenesis inhibition. An Agilent Mouse lncRNA Microarray (design ID: 085631) was used in this experiment. Differentially expressed mRNAs or lncRNAs were identified through fold change and the *P*-values were calculated using the *t*-test. The threshold between upregulated and downregulated RNAs was set as fold change ≥2.0 and *P* < 0.05.

**FIGURE 4:**
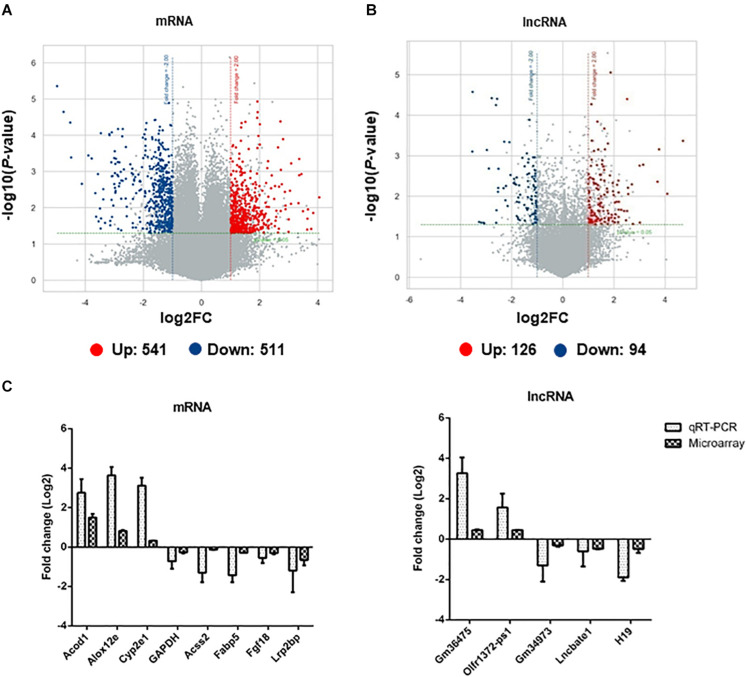
The identification and validation of differentially expressed mRNAs and lncRNAs in adipose tissues of EgPSC infected mice. The volcano plots show the distributions of **(A)** mRNAs and **(B)** lncRNAs. The x-axis indicates the fold change. The horizontal green line represents the filter criterion (threshold *P* ≤ 0.05); red dots, upregulated; blue dots, downregulated. **(C)** qRT-PCR validation of randomly selected differentially expressed mRNAs and lncRNAs from the microarray data.

Among all the mRNAs and lncRNAs detected in the subcutaneous adipose tissues of infected mice, 1052 mRNAs (Supplementary Table S3) and 220 lncRNAs (Supplementary Table S4) were differentially expressed (upregulated: 541 mRNAs, 126 lncRNAs; downregulated: 511 mRNAs, 94 lncRNAs). The volcano plots of differentially expressed mRNAs and lncRNAs are shown in Figures 4A,B, respectively. Red and blue dots indicated significantly upregulated and downregulated transcripts, respectively. The different expression profiles of lncRNAs and mRNAs between control and infected mice indicated that these transcripts may be involved in the differential process of adipose tissues. Moreover, the scatter plots of differentially expressed mRNAs and lncRNAs are shown in Supplementary Figure S5.

To validate the results obtained with microarray analysis, 7 differentially expressed mRNAs (Acod1, Alox12e, Acss2, Fabp5, Fgf18, Lrp2bp, GAPDH) and 5 lncRNAs (Gm36475, Olfr1372-ps1, Gm34973, Lmcbate1, and H19) were randomly selected for qRT-PCR. A consistent expression trend of these genes was observed (Figure 4C).

### GO and KEGG Pathway Analyses of Differentially Expressed mRNAs

**FIGURE 5:**
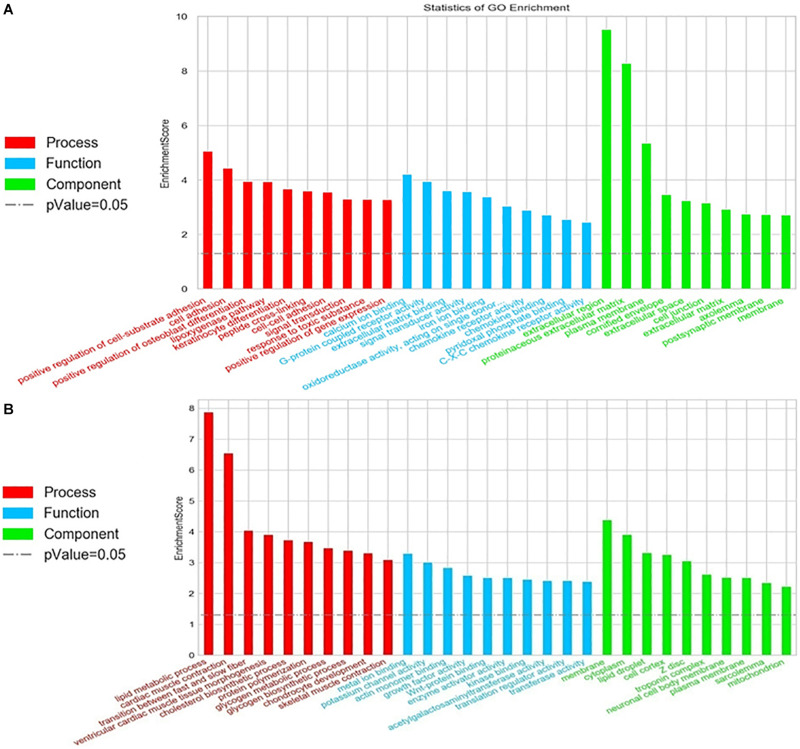
Gene Ontology (GO) analysis of the differentially expressed mRNAs. Go annotation of differentially expressed mRNAs with top 10 enrichment scores covering domains of biological processes, cellular components, and molecular functions. **(A)** Upregulated and **(B)** downregulated mRNAs. The GO terms with corrected *P* < 0.05 were considered significantly enriched by differentially expressed genes.

To evaluate the enrichment of mRNAs in biological processes, cellular components, and molecular functions, Gene Ontology (GO) analysis was performed on 1052 significantly expressed mRNAs noted from the microarray outcome. Based on the biological processes in the gene ontology classification, the differentially expressed mRNAs (fold change ≥2) were classified into different functional categories. Figure 5 shows the GO annotation of upregulated (Figure 5A) and downregulated (Figure 5B) mRNAs with the top 10 enrichment scores covering the three domains. According to GO analysis, upregulated genes were mainly involved in a variety of biological processes, including cell signaling transduction and lipid metabolism, such as “G-protein coupled receptor activity (GO: 0004930)” and “lipoxygenase pathway (GO: 0019372)” Conversely, “lipid metabolic process (GO: 0006629),” “cholesterol biosynthetic process (GO: 0006695),” and “lipid droplet (GO: 0005811)” were downregulated (also listed in Supplementary Table S5). These terms may play a key role in the disorder of lipid metabolism.

**FIGURE 6:**
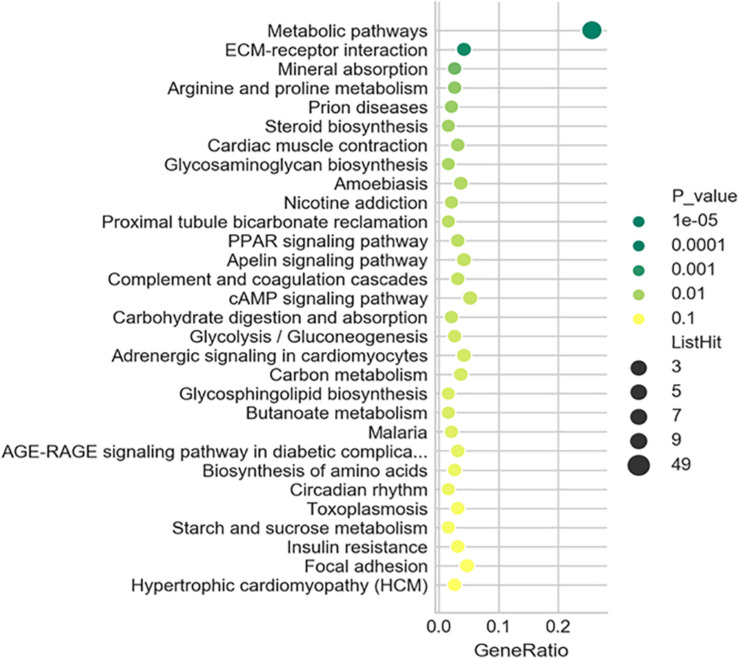
Kyoto Encyclopedia of Genes and Genomes (KEGG) analysis of the differentially expressed mRNAs. Top 30 terms in the pathway analysis of differentially expressed mRNAs after EgPSC infection. The larger the point, the more genes fall into this pathway and the greener point means higher significance of enrichment.

**FIGURE 7:**
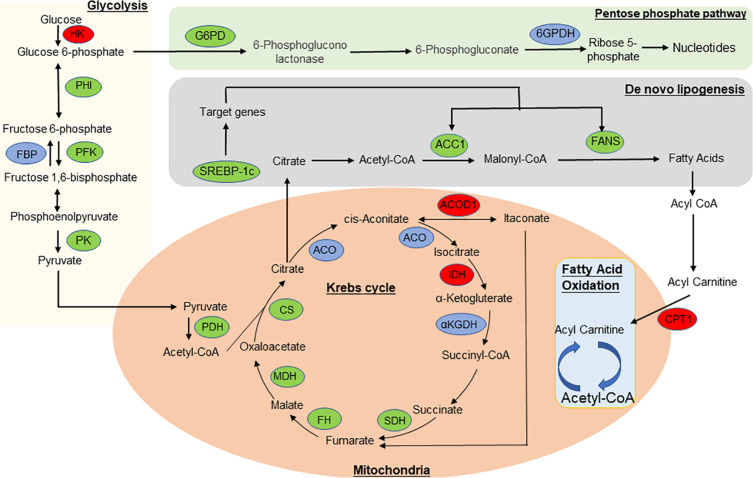
The overview of metabolic reprogramming in adipose tissue in EgPSC infected mice. The metabolism diagram which shows the expression profile of key enzymes. “lipoxygenase” pathways were significantly upregulated while “glycolysis,” “tricarboxylic acid (TCA) cycle,” “pentose phosphate pathway,” and “*de novo* lipogenesis” pathways were dramatically downregulated. Enzymes identified in our study are marked in red oval (upregulated) and green oval (downregulated), while other enzymes are marked in blue oval. HK, hexokinase; PFK, phosphofructokinase; FBP, fructose bisphosphatase; PHI, phosphate isomerase; PK, pyruvate kinase; PDH, pyruvate dehydrogenase complex; G6PD, glucose-6-phosphate dehydrogenase; 6GPDH, 6-phosphogluconate dehydrogenase; CS, citrate synthase; CPT1, carnitine palmitoyltransferase-1; SDH, succinate dehydrogenase; ACO, aconitase; IDH, isocitrate dehydrogenase; αKGDH, α-ketoglutarate dehydrogenase; FH, fumarate hydratase; MDH, malate dehydrogenase; ACOD1, aconitate decarboxylase 1; ACC1, acetyl coenzyme A carboxylase-1; FAS, fatty acid synthase; SREBP-1c, sterol regulatory element-binding protein-1c.

To further understand the predicted biological functions of the aforementioned differentially expressed mRNAs, their pathways and molecular interactions were then predicted by Kyoto Encyclopedia of Genes and Genomes (KEGG) pathway enrichment analysis. The top 30 enriched pathways are shown in Figure 6. Among others (Supplementary Table S2), “Arginine and proline metabolism (mmu00330),” “cyclic adenosine monophosphate (cAMP) signaling pathway (mmu04024),” “carbon metabolism (mmu01200),” and “insulin resistance (mmu04931)” were thought to be closely associated with adipocyte metabolism and deserved further study. Notably, in comparison to the control mice, the expression of enzymes in “glycolysis,” “TCA,” and “*de novo* lipogenesis” pathways were significantly downregulated, while “fatty acid oxidation” was activated after infection (Figure 7 and Supplementary Table S6). This suggested that the EgPSC infection reprogrammed the adipose tissue metabolism.

### lncRNA Transcription Factor Network Analysis

**FIGURE 8:**
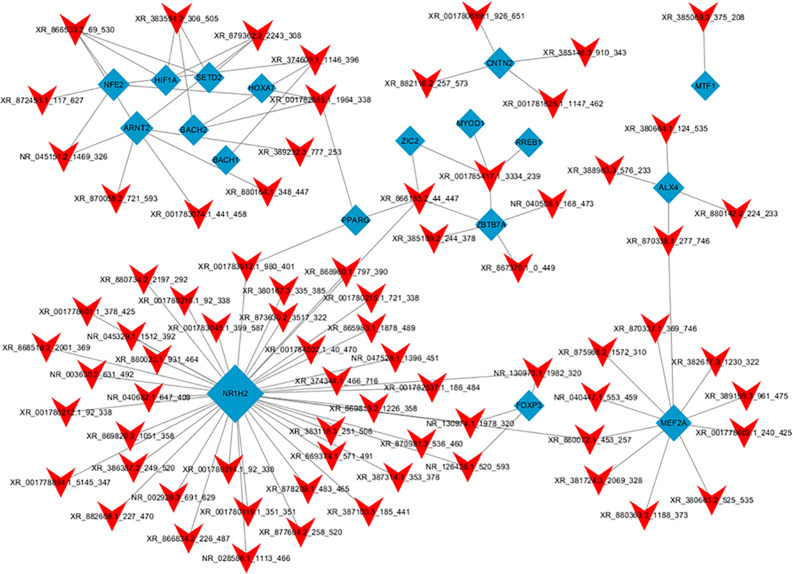
Network of the top 100 most related lncRNA-transcription factor pairs. The co-expression networks of the lncRNA and transcription factor were constructed using Cytoscape software (version 3.5.1). Red arrowheads, lncRNAs; blue diamonds, transcription factors.

Hypergeometric distribution was used to characterize the relationship between transcription factors and differentially expressed lncRNAs. The frequency of each transcription factor (TF) was counted, and the one with more functional annotations was recorded to reflect the overall functional distribution of different lncRNAs. A core network of the top 100 lncRNA-TF pairs was constructed by ranking the *P*-value (Figure 8). These lncRNAs were mostly regulated by contactin 2, metal-regulatory transcription factor 1, aristaless like homeobox 4 (ALX4), aryl hydrocarbon receptor nuclear translocator 2 (ARNT2), and nuclear receptor 1 type H2 (NR1H2). Among these TFs, ARNT2 has been demonstrated to be associated with the prevention of obesity and obesity-related diseases (Swarbrick et al., 2011). Studies have shown that ARNT2 is necessary for the production of secretory hormones, and an induced missense mutation in ARNT2 developed hepatic steatosis and abnormalities in glucose homeostasis (Turer et al., 2018).

### lncRNA Target-TF Network Analysis

**FIGURE 9:**
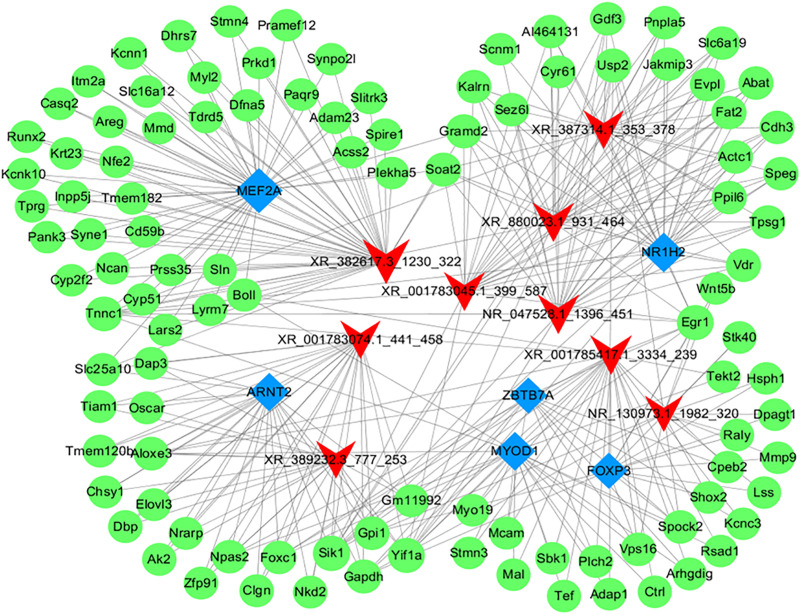
Network of the top 10 most related lncRNA-target-transcription factors. The co-expression networks of the lncRNA, target mRNA and transcription factor were constructed using Cytoscape software (version 3.5.1). Red arrowheads, lncRNAs; green diamonds, mRNAs; blue diamonds, transcription factors.

To further identify the functions of each dysregulated lncRNA, the top 50 differentially expressed lncRNAs and their co-expressed mRNAs pairs were analyzed to conduct the lncRNA-target-TF network (Figure 9). LncRNA NR130973 was predicted to be mainly regulated by these TFs and participates in the Wnt signaling pathway, which has been reported to be related to inflammation and adipose cell function (Kanazawa et al., 2004).

## Discussion

The adipose tissue is an important endocrine organ that can respond differentially to physiological cues or metabolic stress by releasing endocrine factors that regulate diverse processes (Scheja and Heeren, 2019). Abnormal accumulation of body fat is associated with insulin resistance and results in obesity, which is the most frequently observed component of metabolic syndrome (Engin, 2019). Thus, adipocyte differentiation regulators have been considered as possible therapeutic interventions for metabolic diseases (Danforth, 2000; Mota de Sá et al., 2017). Although the role of adipose tissue in parasite infection has been neglected for a long time, some progress has been made in recent years. *Trypanosoma cruzi* (González et al., 2019) and *Nippostrongylus brasiliensis* (Yang et al., 2013) were successively shown to inhibit adipocyte differentiation in obese mice that were fed a high-fat diet, providing new insight into the treatment of metabolic diseases. Moreover, a recent study showed that adipose tissue is a major niche for *T. brucei*, which can adapt its metabolism to the fatty acids that are present in the adipose tissue and improve the host’s fatty acid metabolism (Trindade et al., 2016). Since the adipose tissue lies in a lipid-rich environment and most drugs are hydrophilic, it may pose some limitations to the efficacy of drug treatments for parasitic infections (Tanowitz et al., 2017). Therefore, it is paramount to understand the role and mechanisms of parasitic infection in adipogenesis regulation.

This study has examined the effect of infection with the EgPSC on metabolic measures. EgPSC infection resulted in improved glucose tolerance and showed no obvious change of triglyceride, cholesterol, LDL, HDL, and AST/ALT. When compared with the control group, the infected mice showed a decrease in subcutaneous adipose tissue mass and a reduction in adipocyte size, however, the mass of brown adipose tissue had no obvious change post-infection. As a result, this study focused on the subcutaneous adipose tissues. Meanwhile, there was no statistically significant difference of food intake between control and EgPSC infected mice, which indicated that the mass loss of fat tissue should not result from sickness and poor appetite. The results also showed the existence of a M2-like macrophage in infected adipose tissues, which may promote fat lipolysis (Nguyen et al., 2011).

The present study was the first to address the mRNA and lncRNA expression profiles of adipogenesis in EgPSC-infected mice. Parasitic infection can inhibit the differentiation of subcutaneous adipocytes, which is accompanied by a sharp decline in carbohydrate oxidation and increased fat oxidation. Moreover, microarray analysis revealed that 1052 mRNAs and 220 lncRNAs were differentially expressed in the subcutaneous adipose tissue of infected mice. GO and KEGG analyses showed a metabolic switch in the adipose tissue, which was in agreement with the indirect calorimetry results. Additionally, several differentially expressed mRNAs and lncRNAs were involved in insulin resistance and adipocyte differentiation. Thus, the results provided new insights into the metabolic homeostasis mediated by parasitic infection.

The expression of metabolic enzymes in adipose tissue is strongly related to adipocyte morphology and function (Dahlman et al., 2016) because these proteins control the cellular energy demand by regulating the metabolic pathways. This study found that the expression of key enzymes in “glycolysis,” “TCA cycle,” and “*de novo* lipogenesis” was markedly downregulated, while the expression of enzymes in the “lipoxygenase” pathway was significantly upregulated after EgPSC infection. Acetyl coenzyme A carboxylase 1 (ACC1) and fatty acid synthase (FAS) are key enzymes in adipose synthesis, and play a crucial role in regulating lipid homeostasis (Maier et al., 2010; Fullerton et al., 2013; Wallace et al., 2018; Lally et al., 2019). Sterol regulatory element-binding protein is considered to be a master regulator of lipogenesis (Eberlé et al., 2004) and is able to increase the activity of ACC1 and FAS (Horton et al., 2003a, b). The present study showed that the expression of these proteins was significantly downregulated, which indicated that lipogenesis was inhibited. In contrast, the fatty acid oxidation pathway could be enhanced, since the key enzyme carnitine palmitoyltransferase-1 (CPT1) (McGarry et al., 1983, 1989; Purdom et al., 2018) was significantly downregulated. The downregulation of citrate synthase (CS) and malate dehydrogenase (MDH) indicated that the TCA cycle was weakened, which could contribute to an anti-inflammatory microenvironment in the adipose tissue (Márquez et al., 2019). Through indirect calorimetry, we observed a sharp decline in carbohydrate oxidation (*P* < 0.01) and increased fat oxidation (*P* > 0.05), which agreed with metabolic reprogramming. Additionally, the change of carbohydrate and fat oxidations may be explained by the newly discovered myokine β-aminoisobutyric acid (BAIBA), which induces the adipose tissue transition to a “beige” phenotype and enhances fatty acids oxidation (Tanianskii et al., 2019). Overall, EgPSC infection might regulate gene expression, thereby reprogramming the host’s metabolism to control adipocyte differentiation.

The lncRNAs are important members of the non-coding RNA (ncRNA) family and have been considered as a new class of non-protein regulators in adipocyte biology (Zhao et al., 2018). A total of 220 lncRNAs were found to be differentially expressed, some of which were believed to be closely associated with adipocyte differentiation. For example, *WNT5b* is targeted in the function of signaling pathways that regulate pluripotency in stem cells (Kanazawa et al., 2004), and it was downregulated in our study. The overexpression of the *WNT5b* gene was previously shown to promote adipogenesis and enhance adipocytokine gene expression (Maegawa and Maeda, 2006). Previous studies also substantiated the possible roles of *WNT* genes in conferring susceptibility to type 2 diabetes (Kanazawa et al., 2004; Cao et al., 2017). Moreover, the differentially expressed transcript AKT-2, which is required for adipocyte lipid filling (Sanchez-Gurmaches et al., 2019), is involved in apoptotic pathways (Cinti, 2018). After apoptosis, the adipocytes’ debris are reabsorbed by macrophages, inducing chronic low-grade inflammation and potentially contributing to insulin resistance and type 2 diabetes (Cinti, 2018). In addition, the downregulated lncRNA H19 (gene ID: 14955) was of particular interest. It has been reported that the silencing of H19 inhibits adipogenesis and inflammation response by upregulating miRNA-130b, and the expression of H19 was shown to be upregulated in atherosclerotic patients (Han et al., 2018; Sun et al., 2019). However, the function of these lncRNAs in EgPSC-infected adipose tissue requires further investigation.

Overall, this study proposed that EgPSC infection inhibits adipogenesis along with metabolic reprogramming in the subcutaneous adipose tissue of mice. It has been increasingly recognized that parasite derived excretory-secretory products (ESPs), the key substances employed by helminths to downregulate the host’s anti-infection immunity (Harnett, 2014), are able to regulate adipogenesis and improve insulin sensitivity in a high fat diet-induced obesity model (Hussaarts et al., 2015). Thus, in the future, we will use the ESPs of EgPSC to mock the infection to study whether and how EgPSC alters host metabolism.

## Conclusion

The present study showed that EgPSC infection resulted in a reduction of subcutaneous fat along with a decrease in the size of adipocytes. Consistently, indirect calorimetry revealed the change in energy metabolism that was characterized by a lower CO_2_ production and O_2_ consumption, a sharp decline in carbohydrate oxidation, and a slight increase in fat oxidation. Moreover, several differentially expressed lncRNAs and mRNAs were identified to be associated with adipocyte differentiation, insulin resistance, and metabolic reprogramming. Overall, these findings deepened the understanding of parasite-host interplay, metabolic homeostasis mediated by parasitic infection, and provided novel insight into the mechanism of hypertrophic adipocytes in obesity.

## Data Availability Statement

The microarray data has been deposited into GEO (accession: GSE154979, https://www.ncbi.nlm.nih.gov/geo/query/acc.cgi? acc=GSE154979).

## Ethics Statement

The animal study was reviewed and approved by the Laboratory Animal Welfare and Ethics Committee of Xuzhou Medical University, China [SCXK (Su) 2015-0009].

## Author Contributions

WP, HL, and XY conceived and designed the experiments. YL, JW, TL, YG, and FS performed the experiments. YL, JL, and MD analyzed the data. WP and HL contributed reagents, materials, and analysis tools. YL, WP, and FS wrote the manuscript. All authors contributed to the article and approved the submitted version.

## Conflict of Interest

The authors declare that the research was conducted in the absence of any commercial or financial relationships that could be construed as a potential conflict of interest.
