# Supplementary figures and images for: Microarray Analysis of lncRNA and mRNA Reveals Enhanced Lipolysis Along With Metabolic Remodeling in Mice Infected With Larval Echinococcus granulosus
Source: Front Physiol. 2020 Aug 21;11:1078. doi: 10.3389/fphys.2020.01078 (PMC7472464; doi:10.3389/fphys.2020.01078)

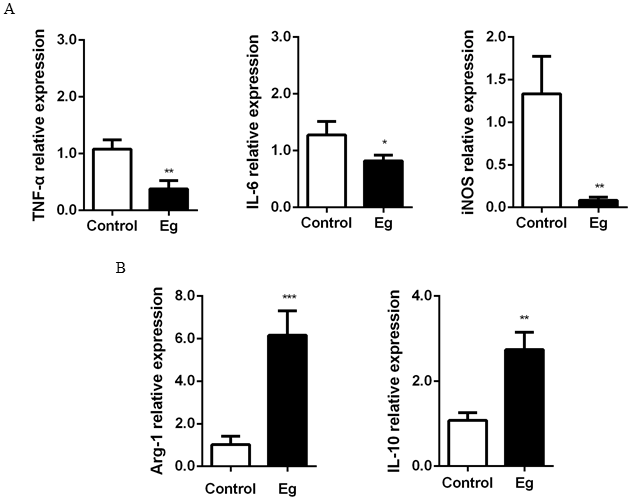

Supplement: Supplementary file 1 [file Image_1.TIF]

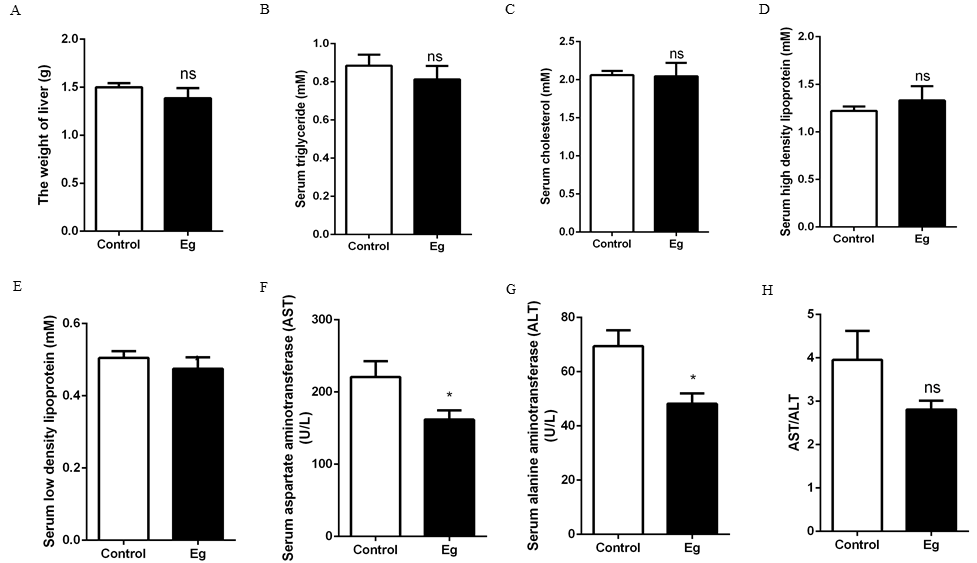

Supplement: Supplementary file 2 [file Image_2.TIF]

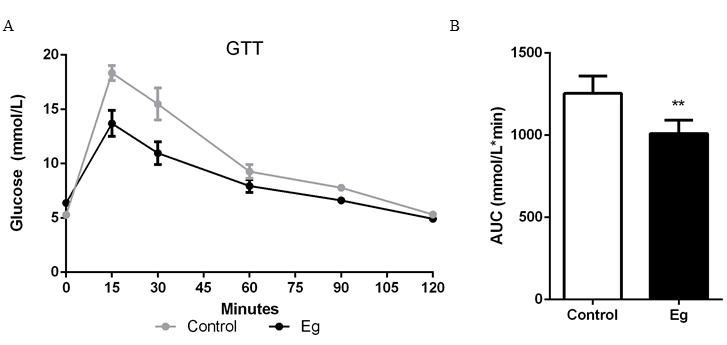

Supplement: Supplementary file 3 [file Image_3.TIF]

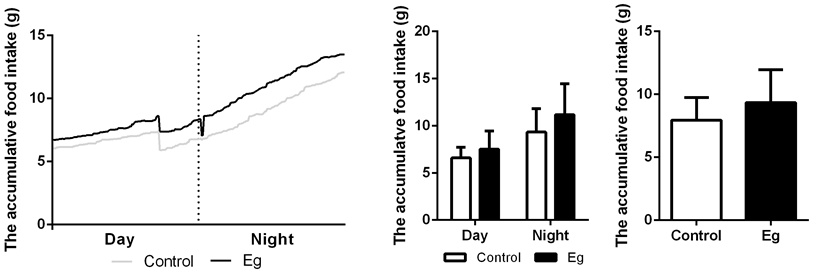

Supplement: Supplementary file 4 [file Image_4.TIF]

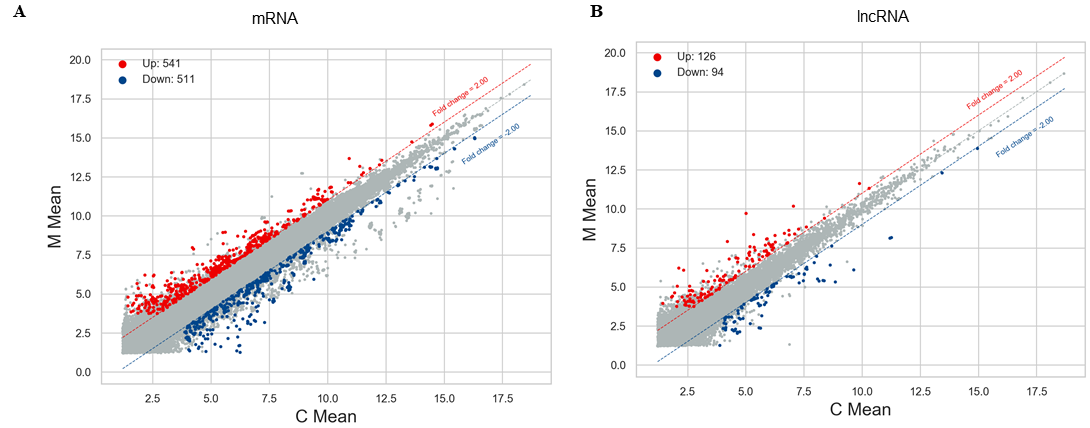

Supplement: Supplementary file 5 [file Image_5.TIF]
